# Standardizing ischemic preconditioning research in applied exercise physiology

**DOI:** 10.1007/s00421-026-06226-5

**Published:** 2026-04-10

**Authors:** Moacir Marocolo, Rodrigo Hohl, Michal Wilk, Gustavo R. Mota, Hiago L. R. Souza

**Affiliations:** 1 Integrated Laboratory of Physiology and Performance (LABIFID), Department of Biophysics and Physiology, Federal University of Juiz de Fora, Juiz de Fora, Minas Gerais Brazil; 2 Institute of Sport Sciences, The Jerzy Kukuczka Academy of Physical Education, Katowice, Poland; 3 Faculty of Physical Education, Gdansk University of Physical Education and Sport, Gdansk, Poland; 4 Exercise Science, Health and Human Performance Research Group, Department of Sport Sciences, Institute of Health Sciences, Federal University of Triângulo Mineiro, Uberaba, Brazil; 5 State University of Minas Gerais, Divinópolis, Brazil

**Keywords:** Ischemia, Performance-enhancing substances, Placebo effect, Research design, Tourniquets

## Abstract

Research on ischemic preconditioning (IPC) within applied exercise physiology has expanded considerably over the past few decades. While IPC demonstrates potential for enhancing exercise performance and accelerating muscle recovery, its widespread adoption is currently hindered by substantial heterogeneity in protocols and applications. Notably, recent evidence suggests that the reported ergogenic effects of IPC become less apparent in studies utilizing a three-arm experimental design (i.e., IPC, placebo and control). This variability compromises reproducibility and complicates the establishment of IPC as a reliable physiological stimulus and an ergogenic aid. Consequently, this narrative review aims to advance the field by identifying gaps, proposing methodological standards and encouraging the standardization of protocol design. We evaluate critical parameters, including cuff width, inflation pressure, number of cycles, and the duration of occlusion and reperfusion, while addressing the implications of experimental design and the confounding influence of placebo effects. As significant questions remain regarding the optimization of IPC for physical work capacity and muscle recovery, a standardized framework is urgently required to guide future research and evidence-based practice in human performance physiology.

## Introduction

Since the 1980s, interventions for ischemia-reperfusion injury have shown significant efficacy in protecting against or minimizing tissue damage (Murry et al. 1986). In physical work capacity, although cuff interventions for occlusion and reperfusion of blood flow before physical exertion have been studied since the 1950s (Muller 1958; Nukada 1955; Collier and Percival 1959), the early 2000s marked a rediscovery of this approach as a method to modulate acute physiological response to exercise (Libonati et al. 2001), called ischemic preconditioning (IPC), which led to a notable increase in publications investigating IPC’s role in enhancing human physical work capacity.

The rediscovery of IPC by applied physiology and exercise researchers has led to a growing body of studies primarily examining cause-and-effect relationships, with exercise performance assessed following IPC across various exercise modalities and/or sport-specific tasks (Incognito et al. 2016). In this context, while some studies have reported improvements in neuromuscular power-dependent tasks (Cheng et al. 2021), aerobic capacity (Crisafulli et al. 2011; da Mota et al. 2019; Bailey et al. 2012; Paull and Van Guilder 2019), and resistance-based tasks (Marocolo et al. 2016a, b; Salagas et al. 2022), other studies have found no improvements in these activities (Gibson et al. 2015; Lalonde and Curnier 2015; Marocolo et al. 2017). In one case, anaerobic performance was even impaired (Paixao et al. 2014).

The experimental designs of the IPC effect on exercise performance exhibit significant methodological heterogeneity, both in terms of IPC protocols and test conditions (O’Brien and Jacobs 2021; Caru et al. 2019). This heterogeneity extends to several technical aspects, including cuff pressure, number of cycles, the duration of occlusion and reperfusion, the interval between IPC and the onset of performance test, among others (Marocolo et al. 2018; Caru et al. 2019; O’Brien and Jacobs 2021). Pressures vary significantly, with some studies applying arbitrary values and others using individual arterial occlusion pressure (AOP) or systolic blood pressure (SBP) as a reference (O’Brien and Jacobs 2021; Caru et al. 2019). The number of IPC cycles also varies substantially, ranging from one (Libonati et al. 2001) to eight (Cocking et al. 2018). Additionally, occlusion and reperfusion durations are inconsistent, generally spanning from two to five minutes (Caru et al. 2019). The interval between IPC and the onset of the performance test also varies considerably, ranging from immediately after IPC to several days (up to seven days) following the final IPC application (O’Brien and Jacobs 2021).

Standardization is vital for reproducibility; however, current evidence suggests a more fundamental concern. Recent meta-analytic findings indicate that purported ergogenic benefits of IPC become less apparent in studies utilizing a three-arm experimental design comprising IPC, placebo and control groups (Souza et al. 2024). This suggests that the lack of standardization and inadequate experimental designs may be masking a dominant placebo effect within IPC interventions.

These technical inconsistencies highlight the complexity of the empirical trial-and-error approach, which has contributed to the historical lack of standardization in IPC protocols (Souza et al. 2024). Given the surge of IPC research in recent decades, there is now sufficient information to harmonize experimental methodology. Therefore, this review provides an expert-informed overview of the current literature, aiming to identify methodological gaps and suggest the standardization of key technical aspects. Such standardization is essential for designing future studies that can accurately differentiate the mechanistic physiological effects of IPC from confounding placebo-perceptual responses.

## Manipulation check: participant expectation and beliefs

A rigorous description of the methodologies employed in IPC studies is essential to the replicability and validity of the results. One often overlooked, but extremely important aspect, is how verbal instructions are provided to participants. A body of evidence has demonstrated that prior verbal instruction can influence performance and exercise outcomes (de la Vega et al. 2017; Hurst et al. 2020; Elhaj et al. 2022). This highlights the significance of managing participants’ expectations regarding IPC cuff intervention through verbal suggestions and its relationship with potential placebo or nocebo effects. Therefore, the detailed description of both the content of the verbal instructions and the identity of those who delivered them is important. This transparency enables a more robust assessment of the potential psychological influence of instructions on participants and contributes to the study’s reliability and reproducibility.

Furthermore, to assess potential placebo response, measurements of participants’ expectations and beliefs about the intervention’s efficacy are strongly encouraged (Beedie et al. 2018). These psychological constructs can significantly influence the results, either amplifying (i.e., placebo effect) or precluding them (i.e., nocebo effect) (Colloca and Barsky 2020) impacting the alleged metabolic and biophysically-mediated IPC effects on physical performance. For instance, previous evidence has demonstrated that varying prior verbal instructions associated with sham-IPC administration can significantly modulate the number of repetitions during knee-extension exercise (Souza et al. 2025a). We therefore suggest that a multi-stage assessment of participant expectations and beliefs may be considered, as these factors can influence performance outcomes. For example, immediately following the IPC or placebo instructions and intervention, but prior to exercise, participants could be asked to rate their anticipated change in exercise performance using a 7-point Likert-type scale, ranging from − 3 (“I expect my performance will be much worse”) to + 3 (“I expect my performance will be much better”), with 0 representing no expected change (Corsi et al. 2019; Fiorio et al. 2014).

To capture the belief in the treatment’s efficacy, participants should complete a 10-cm visual analog scale immediately following the exercise performance protocol. This scale should range from 0 (“The intervention was not effective at all”) to 10 (“The intervention was extremely effective”) (Corsi et al. 2019; Fiorio et al. 2014). By performing these measurements and including them as covariates in data analysis, the large between-subject variability of results and the assumption that IPC responders and non-responders may exist (Incognito et al. 2016), can be addressed.

Prior experience with tourniquets, such as in blood flow restriction training or IPC, is a key methodological consideration that could influence outcomes. Prior exposure may predispose participants to more positive or negative responses to the interventions, with classical conditioning potentially contributing to both placebo and nocebo effects (Colloca and Miller 2011), consequently influencing outcomes. Therefore, prior experience should be carefully assessed and reported. Accordingly, researchers are encouraged to account for prior exposure in study design and analysis.

## Experimental design and the inclusion of a sham-IPC intervention group

To account for the expectation raised by tactile manipulation and subjective expectations, many studies include a sham-IPC intervention. A sham-IPC intervention serves as a placebo control designed to be indistinguishable from the active IPC intervention, typically involving cuff application at a low-pressure that does not achieve vascular occlusion. For standardization purposes, a sham-IPC protocol consisting of three cycles of 5 min of cuff inflation at ~ 20 mmHg, interspersed with 5 min of “reperfusion” (0 mmHg), may be considered and can be applied to either the upper or lower limbs. This approach preserves key features of the intervention (including cuff duration and perceived pressure) while, based on the current evidence, minimizing the likelihood of inducing significant ischemia or altering brachial or popliteal arterial blood flow (Souza et al. 2025b). However, it should be acknowledged that direct evidence from exercise IPC trials remains limited, and this recommendation is partly informed by mechanistic rationale and expert interpretation.

However, it was proposed that sensory feedback during IPC can affect participant perceptions through tactile sensations and trigger bottom-up neurophysiological pathways that may influence cognition and voluntary behavior (Marocolo et al. 2023). Furthermore, it is unclear if perceptions and expectations can be evoked in both IPC and sham-IPC equally, or if the first administration can influence the subsequent administration, even after a washout period. Thus, since it is not possible to blind participants to the cuff pressure applied (Marocolo et al. 2023; O’Brien and Jacobs 2021; Sharma et al. 2015), crossover study designs may introduce expectancy and carryover effects in IPC research.

Accordingly, researchers may consider parallel-group designs, particularly when blinding and perceptual influences are central concerns. For more accurate comparisons across studies, a three-arm parallel experimental design comprising IPC, sham-IPC, and control groups may provide a useful framework to account for prior experience (Beedie et al. 2018; Hurst et al. 2020) and sensory feedback influences on central command (Marocolo et al. 2023), enabling more reliable evidence supporting the peripheral physiological mechanisms underpinning exercise performance changes. Evidence has demonstrated significant placebo responses to low-pressure cuff administration (sham-IPC), similar to those observed with a high-pressure blood-occlusion intervention (Sabino-Carvalho et al. 2017; de Souza et al. 2021a),^,^ highlighting the potential influence of intrinsic psychological or cognitive factors within the IPC context (de Souza et al. 2021b). The absence or inappropriate inclusion of a non-specific placebo group, that is, a control group receiving a sham intervention not specifically designed to mimic the sensory experience of the actual intervention under investigation (e.g., a sham ultrasound), raises concerns regarding the methodological rigor of IPC experiments, particularly with respect to the expectations generated by the intervention. For instance, previous research using sham ultrasound in a within-subject design reported that exercise expectations were significantly different from those observed following IPC (Cheung et al. 2020). Placebo controls are supposed to be indistinguishable from the interventions, except for the active ingredient (Hurst et al. 2020). In this context, we advocate that the active aspect of IPC is the mechanical cycles of local blood flow occlusions interspersed with reperfusions.

Additionally, in a two-arm experimental design, it can be challenging to accurately estimate the intervention’s true effect. For instance, the observed IPC outcomes may be superior to those observed in a control condition, making it difficult to discern whether the effects are attributable to the intervention itself or to other confounding factors, such as placebo responses (Beedie 2007). Consequently, the two-arm design may limit the precision and validity with which the intervention’s efficacy can be evaluated. However, including a third arm and treating the sham condition as an active intervention rather than a passive control enables the assessment of the intervention’s effect, rather than its placebo effect, which can be influenced by cognitive appraisal of the tourniquet manipulation (Marocolo et al. 2023; de Souza et al. 2021b). Moreover, this distinction can only be discerned when the placebo condition is contrasted with a condition lacking experimental manipulation (i.e., control) (Beedie et al. 2018; Hurst et al. 2020). Addressing these psychological factors is crucial for ensuring the accuracy and reliability of the findings in IPC research.

## Standardization of cuff pressure, cuff width and IPC cycles

Despite the extensive body of research on IPC, there remains ambiguity regarding the technical aspects of IPC, such as cuff pressures, cuff width and IPC cycles (Caru et al. 2019; Souza et al. 2024). The literature shows that arbitrary cuff pressures, ranging from 200 to over 300 mmHg, have been used regardless of body size (Souza et al. 2024; O’Brien and Jacobs 2021). In this sense, there is a need for individualized cuff pressure utilization, taking into account individual variability to achieve complete blood flow cessation (i.e., AOP). Proper AOP is considered a fundamental premise underlying IPC-mediated effects, in which humoral or neural pathways are believed to be triggered during occlusion and reperfusion (Marocolo et al. 2025; Sharma et al. 2015; Stokfisz et al. 2017). If complete occlusion is not achieved, the ischemic stimulus may be insufficient, leading to an underestimation of IPC’s ergogenic potential. Conversely, pressure is considered excessively high when it significantly exceeds the AOP. Evidence suggests that an inflation pressure of approximately 130% of AOP serves as an optimal ceiling for occlusion (AORN 2007); this additional percentage provides a necessary safety margin to account for potential blood flow fluctuations (AORN 2007). Beyond this threshold, higher pressures do not enhance the ischemic stimulus but instead elicit localized discomfort and pain (Sharma and Salhotra 2012; AORN 2007), which can confound results through increased sympathetic activation of psychological distress (Marocolo et al. 2023). Utilizing this individualized reference point ensures a consistent physiological stimulus while minimizing the risk of unnecessary pain. To ensure this stimulus is precisely individualized, AOP should be determined using a Doppler stethoscope to monitor the distal pulse during gradual cuff inflation (AORN 2007). Establishing AOP as the minimum cuff pressure required to eliminate arterial flow provides a standardized baseline, allowing for the subsequent application of the + 30% safety margin to ensure consistent ischemia throughout the intervention (AORN 2007).

Research indicates that cuff width is a primary determinant of occlusion efficiency, as wider cuffs distribute pressure over a larger surface area and thus achieve blood flow occlusion at lower pressures than narrower ones (Loenneke et al. 2012; Jessee et al. 2016; Crenshaw et al. 1988). This mechanical advantage is particularly relevant given that pressure tolerance is positively correlated with the volume of tissue involved, where larger limb circumferences typically necessitate higher pressures, which can exacerbate discomfort (Sharma et al. 2014; Sharma and Salhotra 2012; Crenshaw et al. 1988). Despite the heterogeneity reported in IPC protocols (O’Brien and Jacobs 2021), we contend that medium-to-large cuff widths (10–20 cm) are preferable. These dimensions generally allow AOP to be achieved at the minimum effective pressure (Jessee et al. 2016; Loenneke et al. 2012; Sharma et al. 2014; Souza et al. 2025b), thereby mitigating pain perception and reducing the risk of localized tissue damage associated with cuff application (Sharma and Salhotra 2012; AORN 2007). Consistent with previous evidence, a minimum cuff width of ~ 10 cm for upper-limb application (Jessee et al. 2016) and ~ 13.5 cm for lower-limb application may be considered (Loenneke et al. 2012; Souza et al. 2025b). These values are supported by prior work examining cuff characteristics and their influence on arterial occlusion; however, direct evidence from exercise IPC trials remains limited, and these recommendations should be interpreted in light of the available mechanistic data.

According to a recent systematic review, the most frequently reported IPC protocol consist of 3 cycles of 5 min of occlusion interspersed with 5 min of reperfusion (number of cycles ranging from 1 to 4; occlusion duration ranging from 2 to 5 min) (Souza et al. 2024). This protocol aligns with early clinical evidence describing the IPC intervention (Kharbanda et al. 2001) and has been consistently applied in several subsequent clinical studies (Koch et al. 2014; Hausenloy et al. 2007; Enko et al. 2011; Loukogeorgakis et al. 2005). According to the work of Ghosh et al. (2000), IPC efficacy appears to require a specific physiological threshold, with a minimum of 4–5 min of total ischemic stimulus needed to produce maximal protection under in vitro condition. However, once this threshold is exceeded, additional cycles or longer durations do not appear to provide additional benefits. Furthermore, previous evidence demonstrates that increasing the dose by applying more cycles (e.g., 8 cycles of 5 min occlusion interspersed with 5 min of reperfusion) did not yield any further benefit to exercise performance (Cocking et al. 2018). Therefore, the use of 3 cycles of 5 min of occlusion interspersed with 5 min of reperfusion resulting in a total protocol duration of 30 min (15 min of occlusion and 15 min of reperfusion) seems to be sufficient to satisfy the biological requirements for IPC while preventing significantly increases in duration that may compromise the applicability of IPC during acute recovery intervals (e.g., between intermittent high-intensity bouts) or during the pre-exertion preparatory phase.

## Timing between IPC protocols and performance assessments

The optimal timing between the application of IPC protocols and subsequent exercise performance assessment remains an underexplored area within IPC research. Clinical studies have identified 2 primary windows during which IPC effects manifest (Yellon and Downey 2003; Eisen et al. 2004; Koch et al. 2014). The first is a short-term window that begins immediately after the cessation of the cuff intervention and may last approximately 10 min to 4–6 h post-intervention. The second is a long-term window, with effects typically observed starting around 12 to 24 h post-intervention (Koch et al. 2014; Hausenloy and Yellon 2010). In the context of human performance physiology, interventions that elicit short-term effects can be particularly relevant on the pre-exercise preparation phase, within a timeline ranging from hours to minutes before the task (Kilduff et al. 2013). These interventions can be applied moments before the competition, during halftimes, or recovery periods to enhance performance in subsequent periods of play.

It has been postulated that short-term IPC may mediate alterations in hemodynamic responses and muscle oxygen extraction that may justify the benefits of IPC on muscle contractile function (Sharma et al. 2015). Specifically, IPC may enhance red blood cell deformability and induce vasodilation by releasing vasoactive substances, such as nitric oxide (Marocolo et al. 2025; Thijssen et al. 2016). These changes optimize blood flow in microcirculation, facilitating a greater supply of oxygen and nutrients, as well as the removal of CO_2_ and metabolites (Bailey et al. 2012; Heusch 2015; Muller 1958). Regarding the long-term effects, a signaling cascade involving increased mitochondrial activity, reduced oxidative stress, and a decreased inflammatory response is hypothesized, ultimately contributing to the mitigation of exercise-induced muscle damage and the acceleration of the healing process (Marocolo et al. 2025; Sharma et al. 2015).

In addition, bidirectional body-brain integration of muscle sensory feedback to the central command, caused by tactile IPC experience, may delay time to exhaustion by altering perceptions of pain, which influences motor behavior. The mechanistic dual relationship between brain-body integration within the context of IPC is beyond the scope of this study and is well described elsewhere (Marocolo et al. 2023). In this regard, these potential acute psychophysiological and hemodynamic benefits still merit further investigation.

The long-term effects of IPC remain underexplored within applied exercise physiology, with few studies examining physiological or performance outcomes beyond the immediate post-application window. For instance, Williams et al. (2021) reported no significant long-term effects 24 h following an IPC protocol (4 cycles of 5 min of occlusion and reperfusion at AOP), in national and international-level swimmers performing 100-m and 200-m time trials. Similarly, Seeger et al. (2017) found that a comparable protocol (4 cycles of 5 min at 220 mmHg) failed to improve 5-km running time trial performance 24 h post-intervention in moderately to well-trained runners.

In summary, despite the potential for both short and long-term effects, it is essential to consider the context of IPC intervention. Short-term IPC effects appear to contribute more effectively to acute exercise enhancements; consequently, studies should evaluate IPC as a tool for improving performance acutely. Conversely, long-term IPC effects may contribute to recovery following the cessation of activity, mediating the healing process and optimizing recovery. Thus, studies should focus on evaluating the long-term effects of IPC as an intervention to mitigate exercise-induced muscle damage during sports training.

## Influence of training status on the magnitude of IPC-induced performance enhancements

In applied exercise physiology, the participants’ training status is a critical determinant that influences the methodological framework, analysis, and subsequent interpretation of research findings. Training status can significantly modulate the efficacy of interventions (Hopkins et al. 1999) such as IPC, necessitating careful consideration in study design and the evaluation of results. Indeed, previous research indicates that IPC-induced effect sizes fluctuate considerably as a function of the individual’s training status and history (Marocolo et al. 2019). Consequently, researchers have evaluated IPC efficacy across a broad population, ranging from sedentary individuals to highly trained athletes (Souza et al. 2024). The practical significance of these effects is highly context-dependent, while a small-magnitude improvement in sedentary subjects may be of negligible value, an identical effect size in Olympic-level athletes can represent a podium-altering advantage (Buchheit 2016, 2018). Recognizing this distinction is essential for assigning appropriate practical significance to IPC interventions across various populations.

In sedentary or recreationally active individuals, physiological and exercise performance improvements are often more pronounced due to lower baseline fitness levels and a significant potential for adaptation. Consequently, interventions in these populations frequently yield large effect sizes. However, such findings are typically not generalizable to highly trained or elite athletes, whose physiological profiles are closer to their biological ceilings. In these cohorts, athletes may undergo years of rigorous training to achieve only marginal performance increments (Sander et al. 2013; Tonnessen et al. 2015). For Olympic-level athletes, the distinction between success and failure is often determined by exceedingly narrow margins, as performance frequently plateaus despite continued high-volume training (Haugen et al. 2015). Therefore, small effect sizes that appear negligible in less-trained populations can be transformative at the elite level. Enhancements of even a fraction of a percent may determine podium placement. Accordingly, alongside null-hypothesis significance testing, the use of magnitude-based inference can provide more nuanced and valuable insights for optimizing training and pre-exercise preparation phase interventions (Buchheit 2016, 2018).

The structuring of IPC studies should account for these differences by treating participants’ training status as a critical variable. This approach involves stratifying subjects by training status and conducting subgroup analyses to identify differential responses to IPC. Additionally, the interpretation of results should emphasize the context-specific significance of effect sizes, recognizing that smaller magnitudes of improvement in elite athletes can be highly meaningful. The smallest worthwhile change (SWC) approach, typically estimated as a fraction of the between-subject standard deviation (discussed in more detail below), may be relevant for interpreting performance changes across subjects of different fitness levels (Buchheit 2016). This approach will enhance the applicability of research outcomes and contribute to the development of effective IPC protocols tailored to the needs of different athletic populations.

## Considerations for IPC measurements, analysis, and interpretation

The application of IPC as a physiological intervention has demonstrated the potential to enhance acute exercise performance and accelerate muscle recovery during training sessions (Franz et al. 2017; Beaven et al. 2012). The magnitude of these benefits upon a combination of technical parameters and the athlete’s training status, as previously discussed. While repeated IPC application during training sessions may theoretically potentiate recovery compared to single acute bouts, the efficacy of chronic protocols remains a subject of ongoing debate (Banks et al. 2016; Patterson et al. 2021; Lindsay et al. 2017; Slysz and Burr 2019). Conversely, current evidence predominantly supports acute ergogenic benefits, which may attenuate over time (Marocolo et al. 2016a). Furthermore, the observed ergogenic efficacy of IPC is also depend on the precision and sensitivity of the exercise capacity metrics and analytical techniques employed to detect subtle performance changes.

**Fig. 1:**
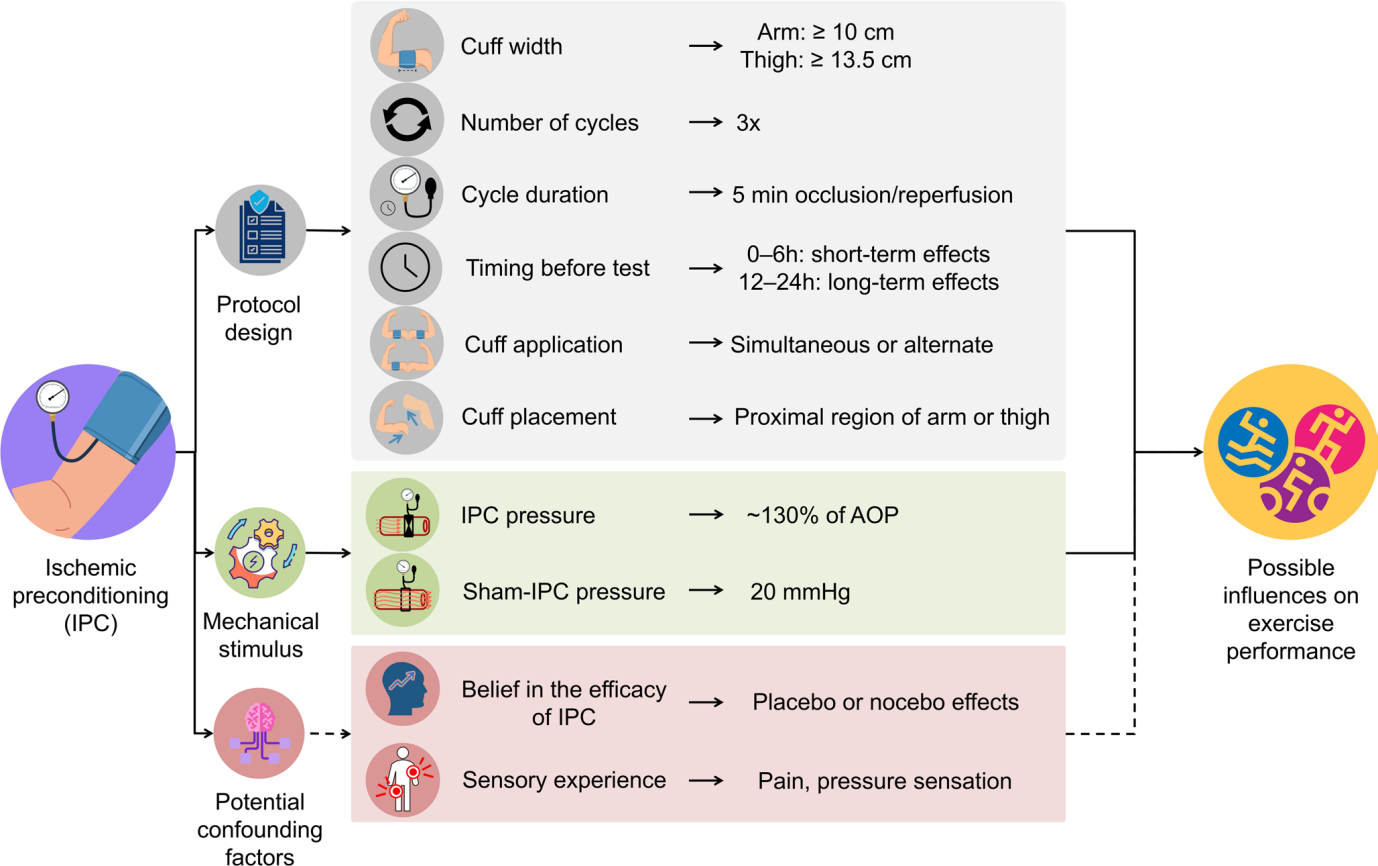
Proposed conceptual framework illustrating IPC protocol parameters and potential confounding factors that may influence exercise performance outcomes. Readers interested in the underlying mechanisms can find a detailed discussion of the potential short- and long-term physiological effects of IPC elsewhere (Marocolo et al. 2025). The dotted line represents factors that may potentially improve or worsen the effects of IPC on exercise performance

Based on the evidence reviewed herein, Fig. 1 presents a conceptual framework of IPC protocol parameter and potential confounding factors, while Table 1 summarizes key methodological domains related to study design, bias control, and reporting practices Furthermore, it is important to adopt an analytical framework tailored to the specific context of athletic performance. Beyond the constraints of traditional null-hypothesis significance testing, complementary approaches that consider the practical relevance of findings, such as effect size estimation and confidence intervals, may provide additional insight. Importantly, the interpretation of results should be anchored in context-specific and biologically meaningful threshold, rather than relying solely on statistical criteria. In this context, SWC has been proposed as a metric to aid the interpretation of exercise performance outcomes, by providing an estimate of the minimal change considered meaningful.

A standard approach, particularly in team sports or group-level analyses, involves utilizing a fraction of the between-subject standard deviation (SD) of the exercise performance metric. For highly trained cohorts, an SWC calculated as 0.2 × SD provides a threshold sensitive enough to detect marginal yet meaningful performance increments. Conversely, for recreationally active subjects or studies involving a broader range of fitness level, a threshold of 0.6 × SD is more appropriate (Buchheit 2016, 2018). This adjustment approach accounts for the greater inter-subject variability typically observed in heterogenous populations.

For instance, consider a group of highly trained sprinters with a mean 100-m time of 12.00 s and a SD of 0.30 s prior to an IPC intervention. Utilizing the standard 0.2 × SD method, the calculated SWC is 0.06 s; therefore, any improvement exceeding this threshold is considered practically meaningful. Under this framework, a runner who improves from 12.10 to 12.02 s (a 0.08-second gain) would surpass the threshold of relevance, whereas an improvement from 11.95 to 11.92 s (a 0.03-second gain) would be classified as trivial. This distinction demonstrates how the SWC identifies practical significance beyond traditional frequentist statistics. By adopting this approach, researchers can more accurately evaluate the impact of IPC, refine interventions to achieve meaningful improvements, and avoid overemphasizing findings that reach statistically significance but lack real-world performance value. However, it should be acknowledged that SWC estimates based solely on statistical distributions may not fully capture biological or practical significance. Therefore, where possible, the use of context-specific or biologically meaningful thresholds (e.g., minimum clinically important differences) may provide a more informative interpretation of performance outcomes.

**Table 1:** Methodological considerations to improve the quality and reporting of IPC research

Topic	Key consideration	Common issues	Practical considerations for future studies
Randomization	Person responsible for random allocation and method used	Inadequate or poorly reported randomization procedures	Clearly describe the method used (e.g., computer-generated randomization) and ensure appropriate sequence generation
Allocation concealment	Mechanism used to implement random group assignment	Lack of reporting or absence of concealment procedures	Use methods such as sealed opaque envelopes or computer-based systems to ensure allocation and reduce selection bias
Blinding feasibility	Ability to blind participants and researchers	Difficulty in blinding due to cuff pressure sensation and discomfort	Consider the use of sham protocols and assess the success of blinding where feasible. In crossover designs, separate staff: one administers conditions (sham/IPC/control) with no involvement in testing or outcomes; another conducts testing blinded to condition. Blind participants to performance data and study hypotheses.
Sham credibility testing	Ability of the sham intervention to replicate key features of IPC	Limited assessment of whether participants believe the sham is real	Include measures of participant beliefs/expectancy to perform manipulation check
Crossover design (carryover effects)	Residual effects between conditions	Insufficient washout periods and lack of carryover assessment	Ensure adequate washout duration and consider testing for carryover effects statistically
Washout period	Time allowed between experimental conditions	Arbitrary or unreported washout durations	Determine washout duration based on physiological rationale and prior literature
Device/Cuff specification	Characteristics of occlusion equipment	Incomplete reporting of cuff width, material, and device type	Provide detailed descriptions (e.g., cuff width, system used, pressure control method) to enhance reproducibility
Preregistration	Registration of study protocol collection in a public repository (e.g., clinicaltrials.gov or similar) prior to data collection	Lack of preregistration and unclear study intentions	Formally record study components (e.g., aims, hypotheses, methods, data analysis strategies, outcomes etc.) to enhance transparency and reduce bias
Selective reporting	Reporting of outcomes and analyses	Incomplete reporting or selective reporting (i.e., “cherry-picking” of results)	Clearly define primary and secondary outcomes a priori and report all planned analyses
Minimal relevant difference	Interpretation of meaningful changes in exercise performance	Focus on statistical significance without considering practical relevance	Incorporate complementary approaches that consider the practical relevance of findings, such as effect size analysis, to distinguish meaningful effects from trivial changes

## Future IPC research directions: innovative proposals

Besides IPC’s potential to improve exercise performance, its protective effects against exercise-induced muscle damage remain poorly investigated. Currently, only few studies have tested the prophylactic effects of IPC in muscle damage (Santos Cerqueira et al. 2021; Patterson et al. 2021; Franz et al. 2018; Page et al. 2017). Future investigations should span both short-term and long-term effects to comprehensively understand the temporal dynamics of IPC’s protective effects.

To enhance the credibility and reproducibility of future IPC studies, it is important to provide an accurate description of research practices. The lack of registered reports in nearly all IPC studies, among other factors, has contributed to an unfavorable assessment of the quality of the current available literature (Souza et al. 2024). Such preregistration helps to ensure greater transparency and accountability in the research process, mitigating questionable research practices such as data exclusion to achieve positive results, manipulation of randomization or selective reporting (Caldwell et al. 2020).

Studies should also expand the currently narrow cause-and-effect relationships in IPC research, focusing on the physiological effects of IPC on central command and peripheral fatigue. Understanding these effects can provide valuable insights into the mechanisms by which IPC may lead to exercise performance improvements. For instance, examining how IPC influences factors such as blood flow, muscle oxygenation, metabolic efficiency, expectations, affective state, cerebral activity, and motor-evoked responses (e.g., V-wave, H-reflex, and M-wave) can help clarify the mechanisms through which IPC might enhance human performance.

Furthermore, when developing experimental protocols, researchers must consider specific and temporal conditions that could influence the outcomes of IPC interventions. For example, the recent history of COVID-19 has introduced new variables into the health status of participants. COVID-19 is associated with an increased risk of thrombosis (Ahmed et al. 2020; Malas et al. 2020), and this could potentially make IPC interventions more dangerous for individuals who have recovered from the disease. Therefore, it is crucial to screen participants for such conditions and to tailor IPC protocols accordingly to ensure participant safety.

## Conclusions

Overall, advancing the field of IPC in applied physiology and exercise necessitates a multifaceted approach, incorporating rigorous methodological designs, standardized protocols and more transparent research practices. Although focused on IPC, these methodological considerations extend to broader challenges in ergogenic-aid research, particularly regarding placebo effects, expectancy, and reproducibility in applied exercise. In this context, improving methodological rigor and standardization is essential not only to reduce bias, but also to enhance reproducibility and allow for a more accurate interpretation of ergogenic effects. By addressing these aspects, future research can provide more convincing evidence on the potential benefits and risks of IPC, ultimately informing better practices for enhancing human performance while ensuring subject safety.

## Abbreviations

AOP: Arterial occlusion pressure
IPC: Ischemic preconditioning
SBP: Systolic blood pressure
SHAM-IPC: Low-pressure cuff administration
SD: Standard deviation
SWC: Smallest worthwhile change

## References

[CR1] Ahmed S, Zimba O, Gasparyan AY (2020) Thrombosis in Coronavirus disease 2019 (COVID-19) through the prism of Virchow’s triad. Clin Rheumatol 39(9):2529–2543. 10.1007/s10067-020-05275-1 32654082 10.1007/s10067-020-05275-1 PMC7353835

[CR2] AORN (2007) Recommended practices for the use of the pneumatic tourniquet in the perioperative practice setting. AORN J 86(4):640–655. 10.1016/j.aorn.2007.09.004 18268836 10.1016/j.aorn.2007.09.004

[CR3] Bailey TG, Jones H, Gregson W, Atkinson G, Cable NT, Thijssen DH (2012) Effect of ischemic preconditioning on lactate accumulation and running performance. Med Sci Sports Exerc 44(11):2084–2089. 10.1249/MSS.0b013e318262cb17 22843115 10.1249/MSS.0b013e318262cb17

[CR4] Banks L, Wells GD, Clarizia NA, Jean-St-Michel E, McKillop AL, Redington AN, McCrindle BW (2016) Short-term remote ischemic preconditioning is not associated with improved blood pressure and exercise capacity in young adults. Appl Physiol Nutr Metab 41(8):903–906. 10.1139/apnm-2016-0024 27439445 10.1139/apnm-2016-0024

[CR5] Beaven CM, Cook CJ, Kilduff L, Drawer S, Gill N (2012) Intermittent lower-limb occlusion enhances recovery after strenuous exercise. Appl Physiol Nutr Metab 37(6):1132–1139. 10.1139/h2012-101 22970789 10.1139/h2012-101

[CR7] Beedie CJ (2007) Placebo effects in competitive sport: qualitative data. J Sports Sci Med 6(1):21–28 24149220 PMC3778695

[CR6] Beedie C, Benedetti F, Barbiani D, Camerone E, Cohen E, Coleman D, Davis A, Elsworth-Edelsten C, Flowers E, Foad A, Harvey S, Hettinga F, Hurst P, Lane A, Lindheimer J, Raglin J, Roelands B, Schiphof-Godart L, Szabo A (2018) Consensus statement on placebo effects in sports and exercise: The need for conceptual clarity, methodological rigour, and the elucidation of neurobiological mechanisms. Eur J Sport Sci 18(10):1383–1389. 10.1080/17461391.2018.1496144 30114971 10.1080/17461391.2018.1496144

[CR8] Buchheit M (2016) The Numbers Will Love You Back in Return-I Promise. Int J Sports Physiol Perform 11(4):551–554. 10.1123/IJSPP.2016-0214 27164726 10.1123/IJSPP.2016-0214

[CR9] Buchheit M (2018) Magnitudes matter more than beetroot juice. Sport Perform Sci Rep 15(1):1–3

[CR10] Caldwell AR, Vigotsky AD, Tenan MS, Radel R, Mellor DT, Kreutzer A, Lahart IM, Mills JP, Boisgontier MP, Consortium for Transparency in Exercise Science C (2020) Moving Sport and Exercise Science Forward: A Call for the Adoption of More Transparent Research Practices. Sports Med 50(3):449–459. 10.1007/s40279-019-01227-1 32020542 10.1007/s40279-019-01227-1

[CR11] Caru M, Levesque A, Lalonde F, Curnier D (2019) An overview of ischemic preconditioning in exercise performance: A systematic review. J Sport Health Sci 8(4):355–369. 10.1016/j.jshs.2019.01.008 31333890 10.1016/j.jshs.2019.01.008 PMC6620415

[CR12] Cheng CF, Kuo YH, Hsu WC, Chen C, Pan CH (2021) Local and remote ischemic preconditioning improves sprint interval exercise performance in team sport athletes. Int J Environ Res Public Health 18(20). 10.3390/ijerph182010653 10.3390/ijerph182010653 PMC8535734 34682399

[CR13] Cheung CP, Slysz JT, Burr JF (2020) Ischemic Preconditioning: Improved Cycling Performance Despite Nocebo Expectation. Int J Sports Physiol Perform 15(3):354–360. 10.1123/ijspp.2019-0290 31188700 10.1123/ijspp.2019-0290

[CR14] Cocking S, Wilson MG, Nichols D, Cable NT, Green DJ, Thijssen DHJ, Jones H (2018) Is There an Optimal Ischemic-Preconditioning Dose to Improve Cycling Performance? Int J Sports Physiol Perform 13(3):274–282. 10.1123/ijspp.2017-0114 28657799 10.1123/ijspp.2017-0114

[CR15] Collier E, Percival C (1959) The working capacity of muscle during reactive hyperemia. Ergonomics 2:116

[CR16] Colloca L, Barsky AJ (2020) Placebo and Nocebo Effects. N Engl J Med 382(6):554–561. 10.1056/NEJMra1907805 32023375 10.1056/NEJMra1907805

[CR17] Colloca L, Miller FG (2011) How placebo responses are formed: a learning perspective. Philos Trans R Soc Lond B Biol Sci 366(1572):1859–1869. 10.1098/rstb.2010.0398 21576143 10.1098/rstb.2010.0398 PMC3130403

[CR18] Corsi N, Emadi Andani M, Sometti D, Tinazzi M, Fiorio M (2019) When words hurt: Verbal suggestion prevails over conditioning in inducing the motor nocebo effect. Eur J Neurosci 50(8):3311–3326. 10.1111/ejn.14489 31209960 10.1111/ejn.14489

[CR19] Crenshaw AG, Hargens AR, Gershuni DH, Rydevik B (1988) Wide tourniquet cuffs more effective at lower inflation pressures. Acta Orthop Scand 59(4):447–451. 10.3109/17453678809149401 3421083 10.3109/17453678809149401

[CR20] Crisafulli A, Tangianu F, Tocco F, Concu A, Mameli O, Mulliri G, Caria MA (2011) Ischemic preconditioning of the muscle improves maximal exercise performance but not maximal oxygen uptake in humans. J Appl Physiol (1985) 111 (2):530–536. 10.1152/japplphysiol.00266.2011 10.1152/japplphysiol.00266.2011 21617078

[CR21] da Mota GR, Willis SJ, Sobral NDS, Borrani F, Billaut F, Millet GP (2019) Ischemic Preconditioning Maintains Performance on Two 5-km Time Trials in Hypoxia. Med Sci Sports Exerc 51(11):2309–2317. 10.1249/MSS.0000000000002049 31169794 10.1249/MSS.0000000000002049

[CR22] de la Vega R, Alberti S, Ruiz-Barquin R, Soos I, Szabo A (2017) Induced beliefs about a fictive energy drink influences 200-m sprint performance. Eur J Sport Sci 17(8):1084–1089. 10.1080/17461391.2017.1339735 28651483 10.1080/17461391.2017.1339735

[CR23] de Souza HLR, Arriel RA, Hohl R, da Mota GR, Marocolo M (2021a) Is Ischemic Preconditioning Intervention Occlusion-Dependent to Enhance Resistance Exercise Performance? J Strength Cond Res 35(10):2706–2712. 10.1519/JSC.0000000000003224 31343550 10.1519/JSC.0000000000003224

[CR24] de Souza HLR, Arriel RA, Mota GR, Hohl R, Marocolo M (2021b) Does ischemic preconditioning really improve performance or it is just a placebo effect? PLoS ONE 16(5):e0250572. 10.1371/journal.pone.0250572 33939730 10.1371/journal.pone.0250572 PMC8092792

[CR25] Eisen A, Fisman EZ, Rubenfire M, Freimark D, McKechnie R, Tenenbaum A, Motro M, Adler Y (2004) Ischemic preconditioning: nearly two decades of research. A comprehensive review. Atherosclerosis 172(2):201–210. 10.1016/S0021-9150(03)00238-7 15019529 10.1016/S0021-9150(03)00238-7

[CR26] Elhaj HM, Imam O, Page BW, Vitale JM, Malek MH (2022) Perceived Consumption of a High-Dose Caffeine Drink Delays Neuromuscular Fatigue. J Strength Cond Res 36(5):1185–1190. 10.1519/JSC.0000000000003932 33370007 10.1519/JSC.0000000000003932

[CR27] Enko K, Nakamura K, Yunoki K, Miyoshi T, Akagi S, Yoshida M, Toh N, Sangawa M, Nishii N, Nagase S, Kohno K, Morita H, Kusano KF, Ito H (2011) Intermittent arm ischemia induces vasodilatation of the contralateral upper limb. J Physiological Sci 61(6):507–513. 10.1007/s12576-011-0172-9 10.1007/s12576-011-0172-9 PMC10718035 21901641

[CR28] Fiorio M, Emadi Andani M, Marotta A, Classen J, Tinazzi M (2014) Placebo-induced changes in excitatory and inhibitory corticospinal circuits during motor performance. J Neurosci 34(11):3993–4005. 10.1523/JNEUROSCI.3931-13.2014 24623777 10.1523/JNEUROSCI.3931-13.2014 PMC6705272

[CR30] Franz A, Behringer M, Nosaka K, Buhren BA, Schrumpf H, Mayer C, Zilkens C, Schumann M (2017) Mechanisms underpinning protection against eccentric exercise-induced muscle damage by ischemic preconditioning. Med Hypotheses 98:21–27. 10.1016/j.mehy.2016.11.008 28012598 10.1016/j.mehy.2016.11.008

[CR29] Franz A, Behringer M, Harmsen JF, Mayer C, Krauspe R, Zilkens C, Schumann M (2018) Ischemic Preconditioning Blunts Muscle Damage Responses Induced by Eccentric Exercise. Med Sci Sports Exerc 50(1):109–115. 10.1249/MSS.0000000000001406 28832392 10.1249/MSS.0000000000001406

[CR31] Ghosh S, Standen NB, Galinanes M (2000) Preconditioning the human myocardium by simulated ischemia: studies on the early and delayed protection. Cardiovasc Res 45(2):339–350. 10.1016/s0008-6363(99)00353-3 10728354 10.1016/s0008-6363(99)00353-3

[CR32] Gibson N, Mahony B, Tracey C, Fawkner S, Murray A (2015) Effect of ischemic preconditioning on repeated sprint ability in team sport athletes. J Sports Sci 33(11):1182–1188. 10.1080/02640414.2014.988741 25517761 10.1080/02640414.2014.988741

[CR33] Haugen T, Tonnessen E, Seiler S (2015) 9.58 and 10.49: nearing the citius end for 100 m? Int J Sports Physiol Perform 10(2):269–272. 10.1123/ijspp.2014-0350 25229725 10.1123/ijspp.2014-0350

[CR35] Hausenloy DJ, Yellon DM (2010) The second window of preconditioning (SWOP) where are we now? Cardiovasc Drugs Ther 24(3):235–254. 10.1007/s10557-010-6237-9 20496105 10.1007/s10557-010-6237-9

[CR34] Hausenloy DJ, Mwamure PK, Venugopal V, Harris J, Barnard M, Grundy E, Ashley E, Vichare S, Di Salvo C, Kolvekar S, Hayward M, Keogh B, MacAllister RJ, Yellon DM (2007) Effect of remote ischaemic preconditioning on myocardial injury in patients undergoing coronary artery bypass graft surgery: a randomised controlled trial. Lancet 370(9587):575–579. 10.1016/S0140-6736(07)61296-3 17707752 10.1016/S0140-6736(07)61296-3

[CR36] Heusch G (2015) Molecular basis of cardioprotection: signal transduction in ischemic pre-, post-, and remote conditioning. Circ Res 116(4):674–699. 10.1161/CIRCRESAHA.116.305348 25677517 10.1161/CIRCRESAHA.116.305348

[CR37] Hopkins WG, Hawley JA, Burke LM (1999) Design and analysis of research on sport performance enhancement. Med Sci Sports Exerc 31(3):472–485. 10.1097/00005768-199903000-00018 10188754 10.1097/00005768-199903000-00018

[CR38] Hurst P, Schipof-Godart L, Szabo A, Raglin J, Hettinga F, Roelands B, Lane A, Foad A, Coleman D, Beedie C (2020) The Placebo and Nocebo effect on sports performance: A systematic review. Eur J Sport Sci 20(3):279–292. 10.1080/17461391.2019.1655098 31414966 10.1080/17461391.2019.1655098

[CR39] Incognito AV, Burr JF, Millar PJ (2016) The Effects of Ischemic Preconditioning on Human Exercise Performance. Sports Med 46(4):531–544. 10.1007/s40279-015-0433-5 26645697 10.1007/s40279-015-0433-5

[CR40] Jessee MB, Buckner SL, Dankel SJ, Counts BR, Abe T, Loenneke JP (2016) The Influence of Cuff Width, Sex, and Race on Arterial Occlusion: Implications for Blood Flow Restriction Research. Sports Med 46(6):913–921. 10.1007/s40279-016-0473-5 26820301 10.1007/s40279-016-0473-5

[CR41] Kharbanda RK, Peters M, Walton B, Kattenhorn M, Mullen M, Klein N, Vallance P, Deanfield J, MacAllister R (2001) Ischemic preconditioning prevents endothelial injury and systemic neutrophil activation during ischemia-reperfusion in humans in vivo. Circulation 103(12):1624–1630 11273988 10.1161/01.cir.103.12.1624

[CR42] Kilduff LP, Finn CV, Baker JS, Cook CJ, West DJ (2013) Preconditioning strategies to enhance physical performance on the day of competition. Int J Sports Physiol Perform 8(6):677–681 23689163 10.1123/ijspp.8.6.677

[CR43] Koch S, Della-Morte D, Dave KR, Sacco RL, Perez-Pinzon MA (2014) Biomarkers for ischemic preconditioning: finding the responders. J Cereb Blood Flow Metab 34(6):933–941. 10.1038/jcbfm.2014.42 24643082 10.1038/jcbfm.2014.42 PMC4050240

[CR44] Lalonde F, Curnier DY (2015) Can anaerobic performance be improved by remote ischemic preconditioning? J Strength Cond Res 29(1):80–85. 10.1519/JSC.0000000000000609 25068802 10.1519/JSC.0000000000000609

[CR45] Libonati JR, Howell AK, Incanno NM, Pettee KK, Glassberg HL (2001) Brief muscle hypoperfusion/hyperemia: an ergogenic aid? J Strength Cond Res 15(3):362–366 11710666

[CR46] Lindsay A, Petersen C, Blackwell G, Ferguson H, Parker G, Steyn N, Gieseg SP (2017) The effect of 1 week of repeated ischaemic leg preconditioning on simulated Keirin cycling performance: a randomised trial. BMJ Open Sport Exerc Med 3(1):e000229. 10.1136/bmjsem-2017-000229 28761713 10.1136/bmjsem-2017-000229 PMC5530127

[CR47] Loenneke JP, Fahs CA, Rossow LM, Sherk VD, Thiebaud RS, Abe T, Bemben DA, Bemben MG (2012) Effects of cuff width on arterial occlusion: implications for blood flow restricted exercise. Eur J Appl Physiol 112(8):2903–2912. 10.1007/s00421-011-2266-8 22143843 10.1007/s00421-011-2266-8 PMC4133131

[CR48] Loukogeorgakis SP, Panagiotidou AT, Broadhead MW, Donald A, Deanfield JE, MacAllister RJ (2005) Remote ischemic preconditioning provides early and late protection against endothelial ischemia-reperfusion injury in humans: role of the autonomic nervous system. J Am Coll Cardiol 46(3):450–456. 10.1016/j.jacc.2005.04.044 16053957 10.1016/j.jacc.2005.04.044

[CR49] Malas MB, Naazie IN, Elsayed N, Mathlouthi A, Marmor R, Clary B (2020) Thromboembolism risk of COVID-19 is high and associated with a higher risk of mortality: A systematic review and meta-analysis. EClinicalMedicine 29:100639. 10.1016/j.eclinm.2020.100639 33251499 10.1016/j.eclinm.2020.100639 PMC7679115

[CR53] Marocolo M, Marocolo IC, da Mota GR, Simao R, Maior AS, Coriolano HJ (2016a) Beneficial Effects of Ischemic Preconditioning in Resistance Exercise Fade Over Time. Int J Sports Med 37(10):819–824. 10.1055/s-0042-109066 27348720 10.1055/s-0042-109066

[CR56] Marocolo M, Willardson JM, Marocolo IC, da Mota GR, Simao R, Maior AS (2016b) Ischemic Preconditioning and Placebo Intervention Improves Resistance Exercise Performance. J Strength Cond Res 30(5):1462–1469. 10.1519/JSC.0000000000001232 26466134 10.1519/JSC.0000000000001232

[CR50] Marocolo IC, da Mota GR, Londe AM, Patterson SD, Barbosa Neto O, Marocolo M (2017) Acute ischemic preconditioning does not influence high-intensity intermittent exercise performance. PeerJ 5:e4118. 10.7717/peerj.4118 29204325 10.7717/peerj.4118 PMC5712465

[CR51] Marocolo M, Billaut F, da Mota GR (2018) Ischemic Preconditioning and Exercise Performance: An Ergogenic Aid for Whom? Front Physiol 9:1874. 10.3389/fphys.2018.01874 30622484 10.3389/fphys.2018.01874 PMC6308393

[CR54] Marocolo M, Simim MAM, Bernardino A, Monteiro IR, Patterson SD, da Mota GR (2019) Ischemic preconditioning and exercise performance: shedding light through smallest worthwhile change. Eur J Appl Physiol 119(10):2123–2149. 10.1007/s00421-019-04214-6 31451953 10.1007/s00421-019-04214-6

[CR52] Marocolo M, Hohl R, Arriel RA, Mota GR (2023) Ischemic preconditioning and exercise performance: are the psychophysiological responses underestimated? Eur J Appl Physiol 123(4):683–693. 10.1007/s00421-022-05109-9 36478078 10.1007/s00421-022-05109-9

[CR55] Marocolo M, Souza HLR, Surke P, Ferrauti A (2025) Potential Short- and Long-Term Physiological Effects of Ischemic Preconditioning as an Ergogenic Aid: Revisiting Foundational Mechanisms and Applications. Sports Med. 10.1007/s40279-025-02232-3 40397368 10.1007/s40279-025-02232-3 PMC12296812

[CR57] Muller EA (1958) Muscular work and muscular blood circulation in reactive hyperemia. Pflugers Arch Gesamte Physiol Menschen Tiere 265(5):29–39. 10.1007/BF00369771 13553716 10.1007/BF00369771

[CR58] Murry CE, Jennings RB, Reimer KA (1986) Preconditioning with ischemia: a delay of lethal cell injury in ischemic myocardium. Circulation 74(5):1124–1136 3769170 10.1161/01.cir.74.5.1124

[CR59] Nukada A (1955) Muscular performance in reactive hyperemia of muscles. Int Z Angew Physiol 16(1):81–82 13306373

[CR60] O’Brien L, Jacobs I (2021) Methodological Variations Contributing to Heterogenous Ergogenic Responses to Ischemic Preconditioning. Front Physiol 12:656980. 10.3389/fphys.2021.656980 33995123 10.3389/fphys.2021.656980 PMC8117357

[CR61] Page W, Swan R, Patterson SD (2017) The effect of intermittent lower limb occlusion on recovery following exercise-induced muscle damage: A randomized controlled trial. J Sci Med Sport 20(8):729–733. 10.1016/j.jsams.2016.11.015 28153608 10.1016/j.jsams.2016.11.015

[CR62] Paixao RC, da Mota GR, Marocolo M (2014) Acute effect of ischemic preconditioning is detrimental to anaerobic performance in cyclists. Int J Sports Med 35(11):912–915. 10.1055/s-0034-1372628 24863728 10.1055/s-0034-1372628

[CR63] Patterson SD, Swan R, Page W, Marocolo M, Jeffries O, Waldron M (2021) The effect of acute and repeated ischemic preconditioning on recovery following exercise-induced muscle damage. J Sci Med Sport 24(7):709–714. 10.1016/j.jsams.2021.02.012 33648866 10.1016/j.jsams.2021.02.012

[CR64] Paull EJ, Van Guilder GP (2019) Remote ischemic preconditioning increases accumulated oxygen deficit in middle-distance runners. J Appl Physiol (1985) 126(5):1193–1203. 10.1152/japplphysiol.00585.2018 30653416 10.1152/japplphysiol.00585.2018

[CR65] Sabino-Carvalho JL, Lopes TR, Obeid-Freitas T, Ferreira TN, Succi JE, Silva AC, Silva BM (2017) Effect of Ischemic Preconditioning on Endurance Performance Does Not Surpass Placebo. Med Sci Sports Exerc 49(1):124–132. 10.1249/MSS.0000000000001088 27580156 10.1249/MSS.0000000000001088

[CR66] Salagas A, Tsoukos A, Terzis G, Paschalis V, Katsikas C, Krzysztofik M, Wilk M, Zajac A, Bogdanis GC (2022) Effectiveness of either short-duration ischemic pre-conditioning, single-set high-resistance exercise, or their combination in potentiating bench press exercise performance. Front Physiol 13:1083299. 10.3389/fphys.2022.1083299 36589445 10.3389/fphys.2022.1083299 PMC9797974

[CR67] Sander A, Keiner M, Wirth K, Schmidtbleicher D (2013) Influence of a 2-year strength training programme on power performance in elite youth soccer players. Eur J Sport Sci 13(5):445–451. 10.1080/17461391.2012.742572 24050460 10.1080/17461391.2012.742572

[CR68] Santos Cerqueira M, Kovacs D, Martins de Franca I, Pereira R, da Nobrega Neto SB, Aires Nonato RD, De Araujo Moura Lemos TM, De Brito Vieira WH (2021) Effects of Individualized Ischemic Preconditioning on Protection Against Eccentric Exercise-Induced Muscle Damage: A Randomized Controlled Trial. Sports Health 13(6):554–564. 10.1177/1941738121995414 33622116 10.1177/1941738121995414 PMC8558991

[CR69] Seeger JPH, Timmers S, Ploegmakers DJM, Cable NT, Hopman MTE, Thijssen DHJ (2017) Is delayed ischemic preconditioning as effective on running performance during a 5km time trial as acute IPC? J Sci Med Sport 20(2):208–212. 10.1016/j.jsams.2016.03.010 27260003 10.1016/j.jsams.2016.03.010

[CR70] Sharma JP, Salhotra R (2012) Tourniquets in orthopedic surgery. Indian J Orthop 46(4):377–383. 10.4103/0019-5413.98824 22912509 10.4103/0019-5413.98824 PMC3421924

[CR71] Sharma V, Cunniffe B, Verma AP, Cardinale M, Yellon D (2014) Characterization of acute ischemia-related physiological responses associated with remote ischemic preconditioning: a randomized controlled, crossover human study. Physiol Rep 2(11):e12200. 10.14814/phy2.12200 25413320 10.14814/phy2.12200 PMC4255807

[CR72] Sharma V, Marsh R, Cunniffe B, Cardinale M, Yellon DM, Davidson SM (2015) From Protecting the Heart to Improving Athletic Performance - the Benefits of Local and Remote Ischaemic Preconditioning. Cardiovasc Drugs Ther 29(6):573–588. 10.1007/s10557-015-6621-6 26477661 10.1007/s10557-015-6621-6 PMC4674524

[CR73] Slysz JT, Burr JF (2019) Impact of 8 weeks of repeated ischemic preconditioning on running performance. Eur J Appl Physiol 119(6):1431–1437. 10.1007/s00421-019-04133-6 30953176 10.1007/s00421-019-04133-6

[CR75] Souza HLR, Oliveira GT, Meireles A, Dos Santos MP, Vieira JG, Arriel RA, Patterson SD, Marocolo M (2024) Does ischemic preconditioning enhance sports performance more than placebo or no intervention? A systematic review with meta-analysis. J Sport Health Sci:101010. 10.1016/j.jshs.2024.101010 10.1016/j.jshs.2024.101010 PMC11880722 39536913

[CR74] Souza HLR, Hurst P, Oliveira GT, Meireles A, Arriel RA, Hohl R, Garcia MAC, Marocolo M (2025a) Positive and negative verbal instructions associated with Sham Ischemic preconditioning moderate improvements of knee-extension resistance exercise in trained men. Int J Sports Physiol Perform 1–8. 10.1123/ijspp.2024-0217 10.1123/ijspp.2024-0217 40348391

[CR76] Souza HLR, Wilk M, de Oliveira GT, Bichowska-Pawęska M, Bernardes BP, Dos Prazeres EO, Camilo GB, Hurst P, Marocolo M (2025b) Determining minimum cuff pressure required to reduce arterial blood flow at rest. Sci Rep 15(1):14322. 10.1038/s41598-025-99334-9 40275037 10.1038/s41598-025-99334-9 PMC12022181

[CR77] Stokfisz K, Ledakowicz-Polak A, Zagorski M, Zielinska M (2017) Ischaemic preconditioning - Current knowledge and potential future applications after 30 years of experience. Adv Med Sci 62(2):307–316. 10.1016/j.advms.2016.11.006 28511069 10.1016/j.advms.2016.11.006

[CR78] Thijssen DH, Maxwell J, Green DJ, Cable NT, Jones H (2016) Repeated ischaemic preconditioning: a novel therapeutic intervention and potential underlying mechanisms. Exp Physiol 101(6):677–692. 10.1113/EP085566 26970535 10.1113/EP085566

[CR79] Tonnessen E, Svendsen IS, Olsen IC, Guttormsen A, Haugen T (2015) Performance development in adolescent track and field athletes according to age, sex and sport discipline. PLoS ONE 10(6):e0129014. 10.1371/journal.pone.0129014 26043192 10.1371/journal.pone.0129014 PMC4456243

[CR80] Williams N, Russell M, Cook CJ, Kilduff LP (2021) Effect of Ischemic Preconditioning on Maximal Swimming Performance. J Strength Cond Res 35(1):221–226. 10.1519/JSC.0000000000002485 29389691 10.1519/JSC.0000000000002485

[CR81] Yellon DM, Downey JM (2003) Preconditioning the myocardium: from cellular physiology to clinical cardiology. Physiol Rev 83(4):1113–1151. 10.1152/physrev.00009.2003 14506302 10.1152/physrev.00009.2003

